# Efficient Flotation Separation of Ilmenite and Olivine in a Weak Alkaline Pulp Using a Ternary Combination Collector Centered around Al^3+^

**DOI:** 10.3390/molecules29184379

**Published:** 2024-09-14

**Authors:** Jinhui Li, Hao He, Yanhai Shao, Chenjie Liu, Rui Li, Hongqin Chen, Xiao Meng

**Affiliations:** Faculty of Land Resources Engineering, Kunming University of Science and Technology, Kunming 650093, China; 15096956049@163.com (J.L.); 13158880067@163.com (H.H.); 19967824778@163.com (C.L.); amnike@icloud.com (R.L.); c91507929@outlooc.com (H.C.); mengxiao20211202@163.com (X.M.)

**Keywords:** ilmenite, flotation, separation, adsorption, combined collector

## Abstract

Due to the similar physical and chemical properties of ilmenite and olivine, separating them is challenging. The flotation process, with the use of collectors, is an effective method. In this study, a ternary collector consisting of aluminum ion (III), benzohydroxamic acid (BHA), and sodium oleate (NaOL) was prepared for the flotation separation of ilmenite and olivine. Through micro-flotation experiments, molecular dynamics simulation (MD), density functional theory (DFT), scanning electron microscopy (SEM), X-ray photoelectron spectroscopy (XPS), and time-of-flight secondary ion mass spectrometry (TOF-SIMS) analysis, the synergistic effect between the components of the ternary collector and the adsorption configuration on the surface of ilmenite was investigated. The results revealed that at pH = 8, Al (III), BHA, and NaOL could coordinate and adsorb effectively on the surface of ilmenite, enhancing its floatability for separation from olivine. The adsorption configuration differed from previous reports, showing a co-adsorption of multiple forms on the surface of ilmenite.

## 1. Introduction

Titanium is widely utilized in various fields, such as automotive, aerospace, military, and medical industries, due to its excellent properties [1,2,3]. Titanium-bearing minerals, primarily in the form of ilmenite, pose challenges in separation due to their similar physicochemical properties to associated minerals. Currently, flotation is deemed an effective method for the recovery of complex fine-grained titanium-bearing minerals [4,5,6]. Flotation is a purification technique conducted at the solid–liquid–gas interface, leveraging differences in wetting properties of various materials for separation. Surface modifiers with selective properties are critical in mineral flotation to enhance wetting differences between minerals for effective separation [7,8].

Fatty acid collectors have been extensively used in titanium-bearing mineral flotation due to their strong capturing ability and cost-effectiveness [9,10,11,12,13]. Currently, high-grade easily purifiable titanium-bearing mineral resources are scarce, with titanium-bearing minerals rich in calcium–magnesium minerals being predominant. Separating titanium-bearing minerals from olivine is challenging due to their highly similar surface properties [14,15,16]. Researchers are focusing on developing highly selective collectors for ilmenite as fatty acid collectors have limited selectivity and fail to meet industry demands. Hydroxamic acid collectors have high selectivity, which can shorten the flotation process of ilmenite and reduce the consumption of pH regulator, but their collection performance is weak and difficult to meet the needs of the industry [17,18,19]. Studies have revealed that the adsorption of anionic collectors on titanium-bearing mineral surfaces is hindered by hydration effects, whereas metal ion activators can effectively weaken the hydration layer. Fang et al. first found that lead ion (II) can effectively activate benzohydroxamic acid (BHA) to improve the floatability of ilmenite, and the separation of ilmenite and titanaugite can be achieved by using a complex with a molar ratio of Pb (II)/BHA = 1/3 [20,21]. Cai et al. utilized dodecyl dimethyl betaine (BS-12) to enhance the flotation performance of sodium oleate (NaOL) under highly acidic conditions (pH = 3). When BS-12 is combined with NaOL at a molar ratio of 6:4, it selectively flocculates ilmenite, thereby improving its floatability [22]. Yang et al. studied the synergistic effect of sodium oleate (NaOL) and styrene phosphonic acid (SPA) in ilmenite flotation. The two exhibit complementary effects, leading to more compact adsorption on the surface of ilmenite, thereby increasing the overall adsorption energy [23]. Wu et al. added NaOL as an auxiliary agent to Pb-BHA to separate ilmenite and titanaugite by flotation and achieved a higher recovery rate than Pb-BHA [24].

However, previous reports on ilmenite flotation with combined collectors have largely overlooked the separation of ilmenite and olivine. Since the hydroxylation of the surface sites of ilmenite will hinder the adsorption of anionic collectors [25,26,27], most of the combined collectors can achieve better flotation results only under strong acidic conditions, which increases the difficulty of the actual process. The activation of metal ions under acid-free conditions can effectively reduce the hydration film on the surface of ilmenite; however, the use of lead nitrate poses environmental hazards. In previous studies, we found that aluminum ion (III)-benzohydroxamic acid (Al-BHA) can effectively separate ilmenite from titanaugite. Nonetheless, due to insufficient recovery rates and high dosage requirements, it remains challenging to meet industrial needs [28,29]. Through DFT calculations, it was found that the lowest unoccupied molecular orbital of Al-BHA was more active and easily reacted with nucleophiles [30]. Therefore, in this work, we demonstrate that the ternary combination collector Al-BHA-NaOL (ABN), which is based on the more environmentally friendly Al(III), can effectively separate ilmenite in weak alkaline pulp. Microscopic visualization of the adsorption configuration revealed that the synergistic mechanism differs from previously reported pre-assembled adsorption, instead suggesting a multi-component co-adsorption process.

## 2. Results and Discussion

### 2.1. Microflotation Tests

The composition ratio and concentration of the combined collector in the pulp can affect its collecting and selectivity, while the pulp pH greatly influences flotation performance. Therefore, the effects of Al^3+^, BHA, NaOL dosage, and pulp pH on flotation were investigated through single mineral micro-flotation experiments.

**Figure 1:**
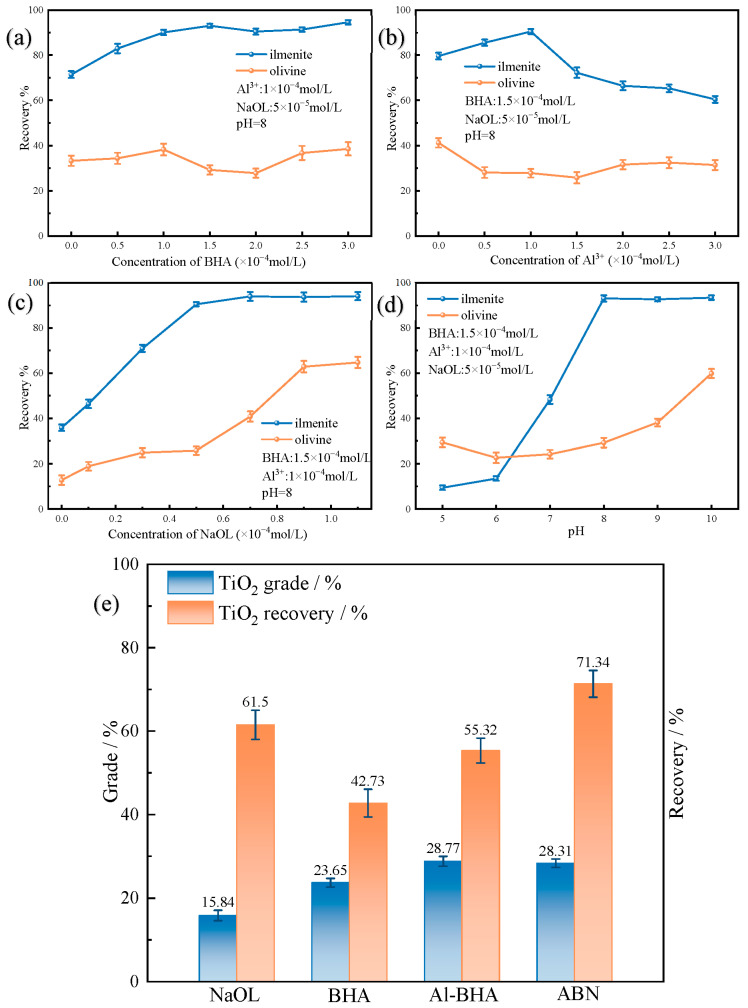
(**a**) The impact of BHA concentration on ilmenite and olivine recovery; (**b**) the influence of Al^3+^ concentration on ilmenite and olivine recovery; (**c**) the effect of NaOL concentration on ilmenite and olivine recovery; (**d**) the impact of pulp pH on ilmenite and olivine recovery; (**e**) flotation of artificial mixed minerals.

Figure 1a demonstrates that a small dosage of BHA results in weak collecting ability and selectivity, with the optimal concentration being 1.5~2 × 10^−4^ mol/L. Figure 1c shows that the optimal concentration of NaOL is 5 × 10^−5^ mol/L. Concentrations of NaOL below 3 × 10^−5^ mol/L lead to a recovery rate of ilmenite below 60%. Excessive NaOL concentration decreases the selectivity of the agent as OL^-^ ions directly adsorb on the surface of olivine, hindering effective separation. Figure 1b shows that the optimal concentration of Al^3+^ is 1 × 10^−4^ mol/L. A small amount of Al^3+^ also has a good activation effect, but the direct interaction of BHA and NaOL with the metal sites on the mineral surface leads to the recovery of olivine up to 40%. Excessive Al^3+^ forms Al hydroxide, leading to competitive adsorption with the collector and reducing the recovery rate of ilmenite. Figure 1d illustrates the impact of pulp pH on recovery rate. Under weak acidic conditions, the recovery rate of ilmenite is less than 40% due to surface dissolution. Weak alkaline conditions result in partial hydroxylation of metal sites on the ilmenite surface, achieving over 90% recovery. A pH > 9 increases the recovery rate of olivine, with ABN adsorbing on the surface and impacting selectivity.

To further verify the flotation performance of the ternary combination collector, an artificial mixed ore was prepared by mixing ilmenite and olivine at a mass ratio of 1:2 for flotation experiments. The experimental conditions were as follows: pH = 8, NaOL concentration = 1.0 × 10^−4^ mol/L, BHA concentration = 3.0 × 10^−4^ mol/L, Al-BHA group with Al^3+^ concentration of 1 × 10^−4^ mol/L and BHA concentration of 3.0 × 10^−4^ mol/L, and ABN group with Al^3+^ concentration of 1 × 10^−4^ mol/L, NaOL concentration of 0.5 × 10^−4^ mol/L, and BHA concentration of 1.5 × 10^−4^ mol/L.

The results indicate that only NaOL has a minimal effect on separating ilmenite and olivine in flotation. BHA shows some separation effect (concentrate TiO_2_ grade = 23.65%), but with a low recovery rate of only 42.73%. Al-BHA can effectively separate the two minerals, yet the recovery rate of 55.32% remains low. ABN can efficiently purify ilmenite (concentrate TiO_2_ grade = 28.01%) with a higher recovery rate of 71.34% (Figure 1e). This suggests that for ilmenite containing calcium and magnesium silicate minerals, the traditional system is ineffective, and ABN ternary collector can achieve efficient recovery and purification of ilmenite under weak alkaline conditions.

### 2.2. Calculation of Self-Assembly Behavior of ABN

#### 2.2.1. MD Simulation of ABN Solution Model

**Figure 2:**
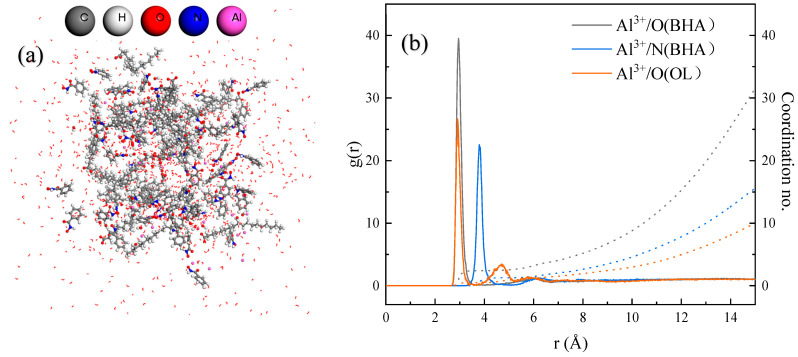
(**a**) ABN aqueous solution model; (**b**) RDF and coordination number.

The self-assembly behavior of ABN ternary collectors in the liquid phase was analyzed using molecular dynamics simulation [31]. In the equilibrium configuration of the ABN aqueous solution simulated by dynamics, most OL molecules are entangled in clusters, with a small amount of BHA molecules free at the cluster’s edge. The majority of Al^3+^ ions are located inside the cluster, binding with OL or BHA. The spatial relationship between the components of ABN was further analyzed by calculating the radial distribution function (RDF) and coordination numbers of Al^3+^ with O (BHA), N (BHA), and O (OL). The results are presented in Figure 2.

The coordination distribution of Al^3+^/O(BHA) starts at 2.6 Å with an average coordination number of 2.4, providing evidence for the five-membered ring configuration of Al-BHA. The N (BHA) around Al^3+^ is distributed around 3.86 Å with an average coordination number of 1.2, indicating no N-Al-O quaternary ring complex is formed. The coordination distance of Al^3+^/O (OL) starts from 2.55 Å with an average coordination number of 0.5, forming an O-Al configuration. The self-assembly reaction in the liquid phase of ABN forms two complexes: BHA-Al-OL and Al-(BHA)_2_.

#### 2.2.2. DFT Calculation

Three chemical reactions are involved in the MD simulation of ABN self-assembly behavior:

BHA^−^ + Al^3+^ → Al(BHA)^2+^

BHA^−^ + BHA^−^ + Al^3+^ → Al(BHA)_2_^+^

BHA^−^ + Al^3+^ + OL^−^ → ABN^+^

The Gibbs free energy of the three reactions was calculated using the DMol3 module to determine the reaction trend. The results are listed in Table 1.

The ΔG_reaction_ values for Al(BHA)^2+^, Al(BHA)_2_^+^, and ABN^+^ are −868.5887 kcal/mol, −1244.3754 kcal/mol, and −1235.2661 kcal/mol at 298.15 K, respectively. The negative values indicate that the reactions can occur at room temperature (298.15 K), and the preparation of ABN only requires pre-mixing at room temperature.

**Figure 3:**
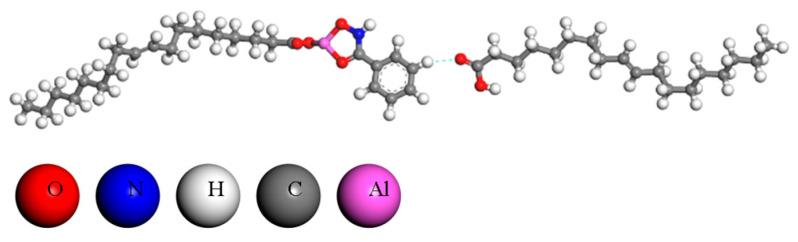
The interaction between ABN and OL.

In addition to chemical bonds and ionic bonds, there are also weak interactions between molecules. By geometric optimization using the DMol3 module, various interactions between the three components were simulated. The results indicate that the C=O of the NaOL molecule forms a hydrogen bond with the benzene ring of BHA, as depicted in Figure 3. This configuration extends the non-polar groups of the collector molecule, thereby enhancing its collection capacity. The interaction energy and charge transfer for this interaction are presented in Table 2.

The results indicate that hydrogen bonds can form between OL and BHA (with a bond length of 2.2392 Å). The charge transfer amount is 0.1082 e, and the interaction energy is −47.8231 kcal/mol. The binding energy is lower than that between water molecules (−6.13 kcal/mol), suggesting that the hydrogen bond between OL-BHA is stronger and more stable in the pulp [32].

**Figure 4:**
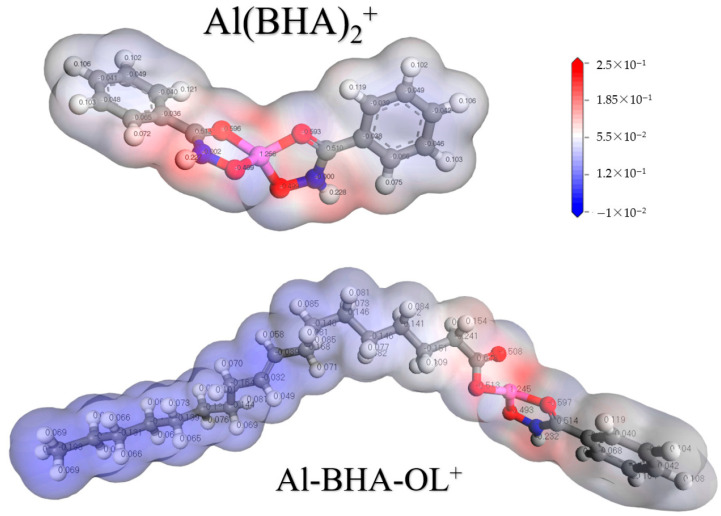
MEP map of Al(BHA)_2_^+^ and Al-BHA-OL^+^.

To predict the chemical active center of the ABN complex, the molecular electrostatic potential (MEP) electronic cloud diagrams of Al(BHA)_2_^+^ and Al-BHA-OL^+^ were calculated (Figure 4). The results reveal that the positive charge distribution of the two molecular ions is centered on the Al atom. The hydrocarbon chain of ABN^+^ is partially neutral, while the O-Al group is positively charged. Therefore, it can easily adsorb onto the negatively charged ilmenite surface with Al serving as a bridge in the slurry.

### 2.3. SEM

**Figure 5:**
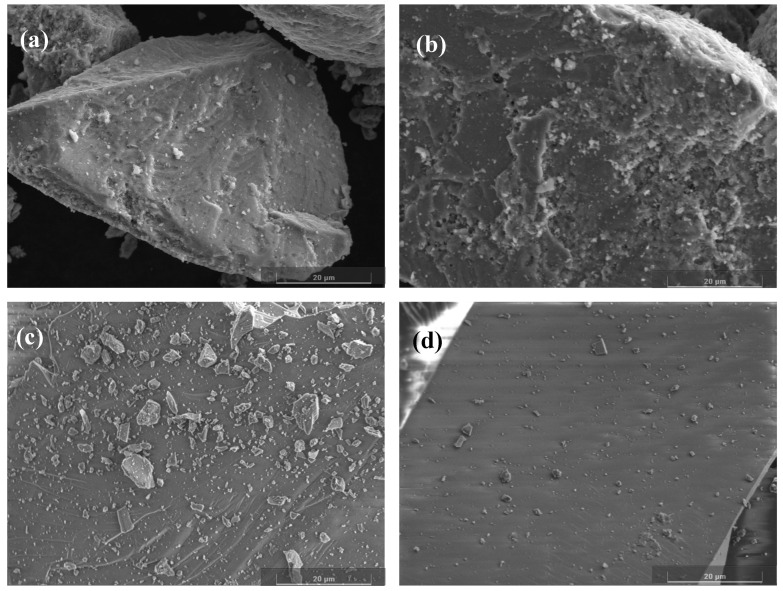
SEM images: (**a**) ilmenite; (**b**) ilmenite + ABN; (**c**) olivine; (**d**) olivine + ABN.

The surface morphology changes of ilmenite and olivine were analyzed using scanning electron microscopy [33,34], and the results are presented in Figure 5. The surface roughness of ilmenite and olivine samples remained similar after natural pH down-slurry treatment (Figure 5a,c). However, when ABN was added at pH = 8, the surface roughness of ilmenite increased, while that of olivine did not change significantly (Figure 5b,d). This difference may be attributed to the adsorption of metal hydroxides and ABN reagents on the ilmenite surface under weak alkaline conditions.

**Figure 6:**
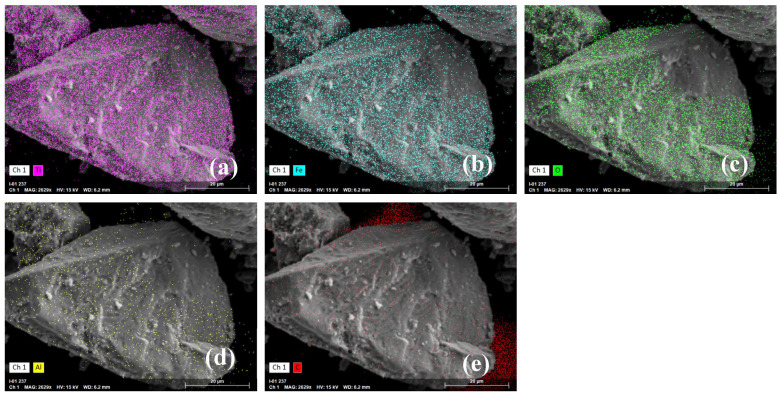
Elemental mapping of ilmenite: (**a**) Ti, (**b**) Fe, (**c**) O, (**d**) Al, (**e**) C.

**Figure 7:**
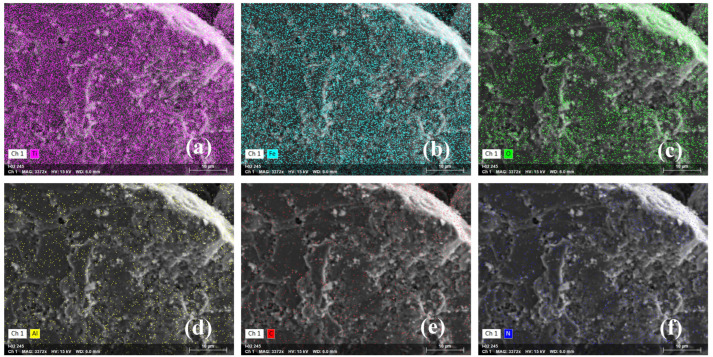
Elemental mapping of ilmenite + ABN: (**a**) Ti, (**b**) Fe, (**c**) O, (**d**) Al, (**e**) C, (**f**) N.

The analysis of surface elements in the samples revealed no significant difference in element distribution on the surface of the olivine sample before and after ABN treatment. Trace amounts of aluminum were detected on the surface of the ilmenite samples, likely due to natural weathering (Figure 6d). Following ABN treatment, the surface content of carbon and aluminum on ilmenite increased, and a small amount of nitrogen was detected, indicating that the ABN component was adsorbed onto the surface of the ilmenite samples (Figure 7d–f).

### 2.4. XPS Analysis

To further analyze the chemical information on the sample surface, XPS analysis was performed on the samples. The results are shown in Figure 7 and Table 3.

**Figure 8:**
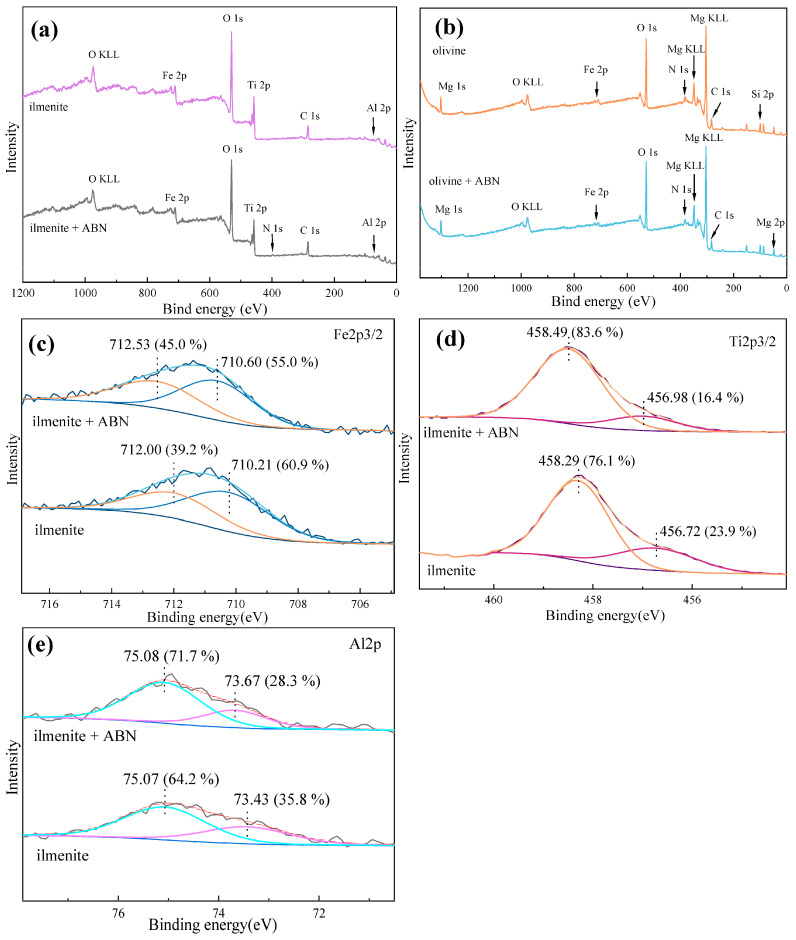
(**a**) XPS full spectrum of ilmenite sample. (**b**) XPS full spectrum of olivine sample. (**c**) Fe2p3/2 high resolution spectra of ilmenite samples. (**d**) Ti2p3/2 high resolution spectra of ilmenite samples. (**e**) Al2p high resolution spectra of ilmenite samples.

No significant N contamination was detected in the pure mineral samples. However, the N 1s peak at 400 eV (Figure 8a) appeared in the ilmenite sample after treatment with ABN, indicating the presence of adsorbate containing BHA. The relative concentration of Al 2p in the pure ilmenite sample was 2.96%, attributed to the introduction of Al element during natural weathering. Following ABN treatment, the relative concentration of Al 2p on the ilmenite surface increased to 3.56%, suggesting adsorption of Al^3+^ on the ilmenite. The C 1s peak was observed at 484.8 eV, with its relative concentration increasing from 22.18% to 25.06% after ABN treatment, confirming further adsorption of flotation reagents on the ilmenite surface. Minimal changes were observed in the surface elements of olivine after ABN treatment, with a slight increase of 0.2% in Al 2p content and 0.4% in O 1s content, indicating possible formation of aluminum hydroxide on the olivine surface. These surface element findings align with SEM results, supporting the selective adsorption of ABN on the ilmenite surface.

In order to further investigate the adsorption mechanism of ABN, high-resolution spectra of Ti 2p, Fe 2p, and Al 2p in different samples were analyzed (Figure 8c–e). Ilmenite exhibits two Fe 2p3/2 peaks at 710.21 eV and 712.00 eV, attributed to spin-orbit coupling where Fe 2p splits into Fe^2+^ and Fe^3+^ peaks [16]. Following treatment with ABN reagent, the two splitting peaks shifted by 0.39 eV and 0.53 eV, respectively, and the peak area ratio of Fe^2+^:Fe^3+^ changed from 60.8:39.2 to 55:45. This indicates a strong chemical reaction between ABN and the Fe site on the surface of ilmenite, leading to a more saturated state of the Fe site on the surface of ilmenite. The Ti 2p peaks of pure ilmenite samples split into two peaks at 456.72 eV and 458.29 eV, corresponding to Ti^3+^ and Ti^4+^, respectively. Upon ABN treatment, the two splitting peaks shifted by 0.26 eV and 0.2 eV, and the peak area ratio changed from 23.9:76.1 to 16.4:83.6. This indicates that ABN chemically adsorbs at the Ti site on the surface of ilmenite. DFT calculations predicted that Al served as the active center of the ABN reagent. Therefore, the high-resolution spectra of Al 2p on the surface of ilmenite before and after reagent treatment were examined (Figure 8d). The Al 2p orbital on the ilmenite surface splits into two peaks at 73.43 eV and 75.07 eV, corresponding to Al-OH and Al-O, respectively. After ABN treatment, the binding energy of the Al-OH peak shifted by 0.24 eV. The shift of the Al-O peak by 0.01 eV may be attributed to instrument error. The ratio of peak areas indicates a reduction in the hydroxide of Al from 35.87% to 28.3%. This suggests that the aluminum element in the Al-O state on the surface of ilmenite is already saturated, and the aluminum element in the adsorbed ABN component exists in the Al-O state, leading to an increase in the relative concentration of Al element but a decrease in the relative concentration of Al-OH.

### 2.5. TOF-SIMS Analysis

**Figure 9:**
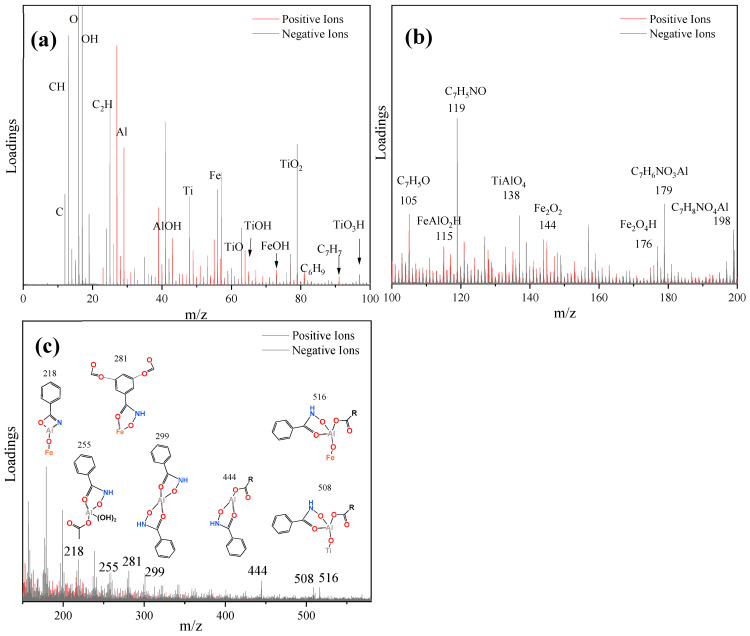
TOF-SIMS mass spectra of ilmenite samples treated by ABN: (**a**) 0–100 *m*/*z*. (**b**) 100–200 *m*/*z*. (**c**) 150–650 *m*/*z*.

The different structural fragments of ABN on the surface of ilmenite were analyzed using TOF-SIMS mass spectrometry. The adsorption configuration of ABN on the ilmenite surface was visually analyzed [35,36]. The TOF-SIMS analysis results of ilmenite samples after ABN treatment are presented in Figure 8. Organic matter fragments were emitted as high-intensity secondary ions, with the fragment information forming a strong signal in the low-mass region (Figure 9a,b). The complex molecular ion exhibited characteristic signals in the 200–600 Da region (Figure 9c). Due to its large molecular weight and weak stability, the molecular ion signal was weak.

Ti, Fe, and hydroxylated Ti(OH) and Fe(OH) sites are simultaneously present on the surface of ilmenite. The fragment information of various BHA and NaOL, as well as the structural fragment information of their bonding with Al, can be obtained from the high-intensity characteristic information in the low-mass region of TOF-SIMS. The formation of Fe-O-Al and Ti-O-Al structures on the surface of Al and ilmenite is confirmed by the characteristic information in the low-quality region (Figure 9a,b). By combining this with the characteristic information in the high-quality region (Figure 9c), the adsorption configuration of ABN on the surface of ilmenite can be inferred.

A small amount of BHA and NaOL are still directly adsorbed on the Fe and Ti sites on the surface of ilmenite, predominantly utilizing Al as a bridge to indirectly interact with the Fe-OH and Ti-OH sites on the surface of ilmenite. The pre-assembled BHA-Al-OL and Al-(BHA)n in ABN adsorb on the surface of ilmenite in the form of Ti-O-Al and Fe-O-Al. Moreover, the C=O group of NaOL also forms hydrogen bonds with the benzene ring of BHA to create larger molecular weight complexes adsorbed on the surface of ilmenite. The Al-BHA-NaOL ternary combination collector contains various active components, exhibiting single-component adsorption, binary complex adsorption, and multi-component complex adsorption on the surface of ilmenite. The complementarity between BHA and NaOL, along with the synergistic effect with Al as the center, enhances its flotation performance significantly.

## 3. Experimental

### 3.1. Materials

The ilmenite and olivine samples utilized in this study were obtained from Panzhihua City, Sichuan Province, China. The ilmenite sample and olivine sample have a purity of 98%, both meeting the experimental requirements. The chemical multi-element analysis of these samples is presented in Table 4.

BHA and NaOL were purchased from Shanghai Macklin Biochemical Co. (Shanghai, China). Aluminum trichloride hexahydrate (AlCl_3_·6H_2_O) was supplied by Tianjin Fengchuan Chemical Reagent Technologies Co. (Tianjin, China). The pH was adjusted using H_2_SO_4_ and NaOH. All reagents were analytical grade reagents, and all experiments were carried out with deionized water (resistivity = 18.25 MΩ × cm).

### 3.2. Microflotation Tests

A XFG5-35 flotation cell (WUHAN EXPLORATION MACHINERY CO., Wuhan, China) and a Plexiglas (WUHAN EXPLORATION MACHINERY CO., Wuhan, China) cell with a volume of 40 mL were used for single mineral flotation tests, with a stirring shaft speed of 1700 rpm. Approximately 2 g of pure mineral powder samples were placed in a tank with 38 mL of deionized water, stirred for 2 min, and then the pH was adjusted with H_2_SO_4_ and NaOH. Flotation reagent was added, stirred for 3 min, manually scraped for 5 min, and the concentrate and tailings were filtered, dried, and weighed. The flotation recovery rate was calculated using the following equation:

R = Mc/(Mc + Mt) × 100% (1)

R is flotation recovery rate, Mc is concentrate quality, and Mt is tailings quality.

Ilmenite and olivine were mixed at a ratio of 0.6 g:1.2 g to simulate ore samples that are difficult to separate. Micro-flotation experiments were carried out in the same way. The grade of TiO_2_ in concentrate products was detected and the recovery rate was calculated using the following formula:

(2) $$R=\frac{Mc\times Gc}{\left(Mc\times Gc+Mt\times Gt\right)}\times 100\%$$

Gc is the concentrate grade and Gt is the tailings grade.

### 3.3. Calculation and Simulation Details

#### 3.3.1. Molecular Dynamics (MD) Simulation

The molecular model was built using Material Studio 2020, and the solution periodic model was created with the Amorphous Cell module. After structural optimization in the universal force field of the Forcite module, molecular dynamics simulation was conducted in the NVT ensemble for a total of 1000 ps with a time step of 1 fs [37,38,39,40,41,42].

#### 3.3.2. Density Functional Theory (DFT) Calculation

The DFT calculation in this paper is completed with the DMol3 module of Materials Studio 2020 software [43,44,45]. The model was established in Materials Studio 2020 software, and the structure was optimized and calculated using DMol3 module in GGA–BLYP functional with cutoff energy of 571.4 eV and k-point of 2 × 2 × 1 [46]. In order to facilitate the convergence of the calculation results, smearing is used to fill the energy levels near the Fermi level according to the thermodynamic distribution. Specific convergence criteria: the energy tolerance is 1.0 × 10^−5^ Ha/atom, the maximum force tolerance is 0.002 Ha/Å, and the maximum displacement tolerance is 0.005 Å.

To investigate the synergistic mechanism of different components in the combined collector, the molecular model is optimized and the energy is calculated using the DMol3 module to predict the thermodynamic properties of the system [47]. The energy of each component and the reaction entropy, enthalpy, and free energy at various temperatures can be determined. Geometric optimization is used to predict the thermodynamic properties and calculate the corrections of enthalpy (H), entropy (S), and free energy (G) at each specified temperature. The correction value of the free energy (G) for each molecular system at a fixed temperature of 298.15 K can be calculated using the following formula:

E_Tcorr_^298.15K^ = E_total_ + G_total_^298.15K^ (3)

The ΔG value (kcal/mol) of the reaction was calculated by the formula:

∆G_reaction_^298.15K^ = [E_tcorr_^298.15K^(product) − E_tcorr_^298.15K^(reactant)] × 627.51 (4)

E_Tcorr_^298.15K^ is the correction value of the system energy at 298.15 K, E_total_ is the total energy of the system, G_total_^298.15K^ is the correction value of the predicted G value at 298.15 K, and ΔG_reaction_^298.15K^ is the free energy of the reaction.

To investigate non-chemical bonding interactions between pharmaceutical components, the model was optimized and calculated using the GGA–PBE functional within the DMol3 module. The role of its molecules in the same system is simulated, and the interaction energy is calculated using the following formula:

E_int_ = E_A-B_ − (E_A_ + E_B_) (5)

E_int_ represents the interaction energy, E_A-B_ represents the total energy of two molecules interacting in the same system, and E_A_ and E_B_ represent the respective energy of the two molecules.

In order to predict the chemical reactivity of the reagent, the molecular ion electrostatic potential was calculated in the GGA–BLYP functional [48].

### 3.4. Scanning Electron Microscopy (SEM)

The surface morphology and composition of ilmenite and forsterite were observed using SEM–EDS. After adsorption, the sample was washed with deionized water to remove loose attachments, dried at 40 °C in a vacuum drying oven, and coated with gold using an ion-coating machine (Longzheng vacuum, Shenzhen, China). Analysis was conducted using a TESCAN CLARA instrument at 15 kV (Männedorf, Switzerland).

### 3.5. X-ray Photoelectron Spectroscopy (XPS) Analysis

At a pH of 8, the flotation reagent was added following the same procedure as in the flotation experiment. After stirring for 5 min, the powder was filtered, washed three times with deionized water, and then dried at 40 °C in a vacuum drying oven to obtain a powder sample for XPS analysis.

XPS measurements were conducted on two mineral samples before and after treatment with reagents using a scanning XPS microprobe system (PHI5000 Versaprobe III XPS, ULVAC-PHI, Chigasaki, Japan). The analysis chamber had a vacuum level of ≤4.78 × 10^−6^ Pa, with a Mono alka X-ray source operating at 1486.6 eV energy, 15 kV voltage, and 4.5 mA beam current.

### 3.6. Time-of-Flight Secondary Ion Mass Spectrometry (TOF-SIMS) Analysis

TOF-SIMS measurements were performed using a PHI nano TOF II Time-of-Flight SIMS instrument (Physical Electronics, Chanhassen, MN, USA) equipped with a Bi_3_^++^ ion source. The parameters were as follows: ion species: Bi_3_^++^, energy: 30 keV, ion current: 2 nA, raster size: 100 μm × 100 μm, mass range: 2~1850 u, mode: high mass resolution mode.

## 4. Conclusions

1. The ternary combined collector ABN holds significant promise in ilmenite flotation due to its ease of preparation, high selectivity, strong collecting ability, multiple active components, and environmental friendliness. This study addresses the research gap regarding highly selective collectors for the separation of ilmenite and olivine under acid-free conditions. Additionally, it offers theoretical guidance for investigating the synergistic effects of combined collectors and for developing new collectors. The ternary combination collector ABN exhibits excellent selective enrichment for ilmenite in weak alkaline conditions (pH = 8). The recovery rate of ilmenite exceeds 90%, whereas the recovery rate of olivine, which shares similar surface properties, is only about 20%.
2. Al^3+^, BHA, and NaOL can undergo self-assembly at room temperature to form BHA-Al-NaOL and Al(BHA)ₙ, with chemical bonds and weak interactions between the components.
3. SEM–EDS and XPS analyses revealed that ABN chemically adsorbs onto Ti and Fe sites on the ilmenite surface, resulting in the deprotonation of the Fe/Ti-OH structure and the formation of Fe-O-Al and Ti-O-Al structures.
4. The TOF-SIMS analysis of the ilmenite surface confirmed the synergistic adsorption configuration of the ternary combination collector. Additionally, it was observed that the pre-assembled reagent adsorption on the mineral surface was not entirely uniform but exhibited various forms of co-adsorption.

## Figures and Tables

**Table 1:** Energy and reaction free energy of each system.

Reactant	BHA^−^	Al^3+^	OL^−^
E_Tcorr_^298.15K^ (Ha)	−475.475364	−240.411413	−855.6647469
Product	Al(BHA)^2+^	Al(BHA)_2_^+^	ABN^+^
E_Tcorr_^298.15K^ (Ha)	−717.2709	−1193.3451	−1573.5211
∆G_reaction_^298.15K^(kcal/mol)	−868.5887	−1244.3754	−1235.2661

**Table 2:** Energy and charge transfer amount.

E(OL)	E(ABN)	E(ABN-OL)	E_int_	Charge Transfer
−855.9163 Ha	−1572.7877 Ha	−2428.7803 Ha	−47.8231 kcal/mol	0.1082 e

**Table 3:** Relative concentration of elements on the ilmenite and olivine surface.

Samples	Element (Mass %)						
	C1s	O1s	Fe2p	Ti2p	Al2p	N1s	
Ilmenite	22.18	57.62	6.99	10.12	2.96	-	
Ilmenite + ABN	25.06	54.91	5.76	9.03	3.56	1.67	
	C1s	O1s	Fe2p	Mg2p	Al2p	N1s	Si2p
Olivine	18.4	52.9	1.7	15.1	0.2	1.6	10.1
Olivine + ABN	18.5	53.3	1.1	15.3	0.6	1.4	9.6

**Table 4:** Chemical multi-element analysis of ilmenite and olivine samples (mass fraction, %).

Sample	TiO_2_	Fe	SiO_2_	CaO	MgO	Al_2_O_3_
Ilmenite	50.12	25.36	1.86	0.21	0.09	0.56
Olivine	0.02	8.47	43.52	0.04	47.23	0.12

## Data Availability

Data are contained within the article.
